# Plio-Pleistocene phylogeography of the Southeast Asian Blue Panchax killifish, *Aplocheilus panchax*

**DOI:** 10.1371/journal.pone.0179557

**Published:** 2017-07-25

**Authors:** Samantha V. Beck, Gary R. Carvalho, Axel Barlow, Lukas Rüber, Heok Hui Tan, Estu Nugroho, Daisy Wowor, Siti Azizah Mohd Nor, Fabian Herder, Zainal A. Muchlisin, Mark de Bruyn

**Affiliations:** 1 Hólar University College, Department of Aquaculture and Fish Biology, Háskólinn á Hólum, Sauðárkrókur, Iceland; 2 Institute of Life and Environmental Sciences, University of Iceland, Reykjavík, Iceland; 3 Molecular Ecology and Fisheries Genetics Laboratory, School of Biological Sciences, Environment Centre Wales, Bangor University, Bangor, United Kingdom; 4 Institute for Biochemistry and Biology, University of Potsdam, Karl-Liebknecht-Strasse, Potsdam (Golm), Germany; 5 Naturhistorisches Museum der Burgergemeinde Bern, Bernastrasse, Bern, Switzerland; 6 Institute of Ecology and Evolution, University of Bern, Baltzerstrasse, Bern, Switzerland; 7 Lee Kong Chian Natural History Museum, National University of Singapore, Singapore; 8 Indonesian Research Institute for Freshwater Aquaculture, Bogor, Java, Indonesia; 9 Research Center for Biology (Puslit Biologi), Indonesian Institute of Sciences (LIPI), Cibinong, Indonesia; 10 School of Biological Sciences, Universiti Sains Malaysia, Penang, Malaysia; 11 Sektion Ichthyologie, Zoologisches Forschungsmuseum Alexander Koenig, Adenauerallee, Bonn, Germany; 12 Department of Aquaculture, Marine & Fishery Sciences, Syiah Kuala University, Banda Aceh, Indonesia; 13 School of Life and Environmental Sciences, University of Sydney, Sydney, New South Wales, Australia; National Cheng Kung University, TAIWAN

## Abstract

The complex climatic and geological history of Southeast Asia has shaped this region’s high biodiversity. In particular, sea level fluctuations associated with repeated glacial cycles during the Pleistocene both facilitated, and limited, connectivity between populations. In this study, we used data from two mitochondrial and three anonymous nuclear markers to determine whether a fresh/brackish water killifish, *Aplocheilus panchax*, Hamilton, 1822, could be used to further understand how climatic oscillations and associated sea level fluctuations have shaped the distribution of biota within this region, and whether such patterns show evidence of isolation within palaeodrainage basins. Our analyses revealed three major mitochondrial clades within *A*. *panchax*. The basal divergence of *A*. *panchax* mitochondrial lineages was approximately 3.5 Ma, whilst the subsequent divergence timings of these clades occurred early Pleistocene (~2.6 Ma), proceeding through the Pleistocene. Continuous phylogeographic analysis showed a clear west-east dispersal followed by rapid radiation across Southeast Asia. Individuals from Krabi, just north of the Isthmus of Kra, were more closely related to the Indian lineages, providing further evidence for a freshwater faunal disjunction at the Isthmus of Kra biogeographic barrier. Our results suggest that Sulawesi, across the Wallace Line, was colonised relatively recently (~30 ka). Nuclear DNA is less geographically structured, although Mantel tests indicated that nuclear genetic distances were correlated with geographic proximity. Overall, these results imply that recent gene flow, as opposed to historical isolation, has been the key factor determining patterns of nuclear genetic variation in *A*. *panchax*, however, some evidence of historical isolation is retained within the mitochondrial genome. Our study further validates the existence of a major biogeographic boundary at the Kra Isthmus, and also demonstrates the use of widely distributed fresh/brackishwater species in phylogeographic studies, and their ability to disperse across major marine barriers in relatively recent time periods.

## Introduction

**Fig 1:**
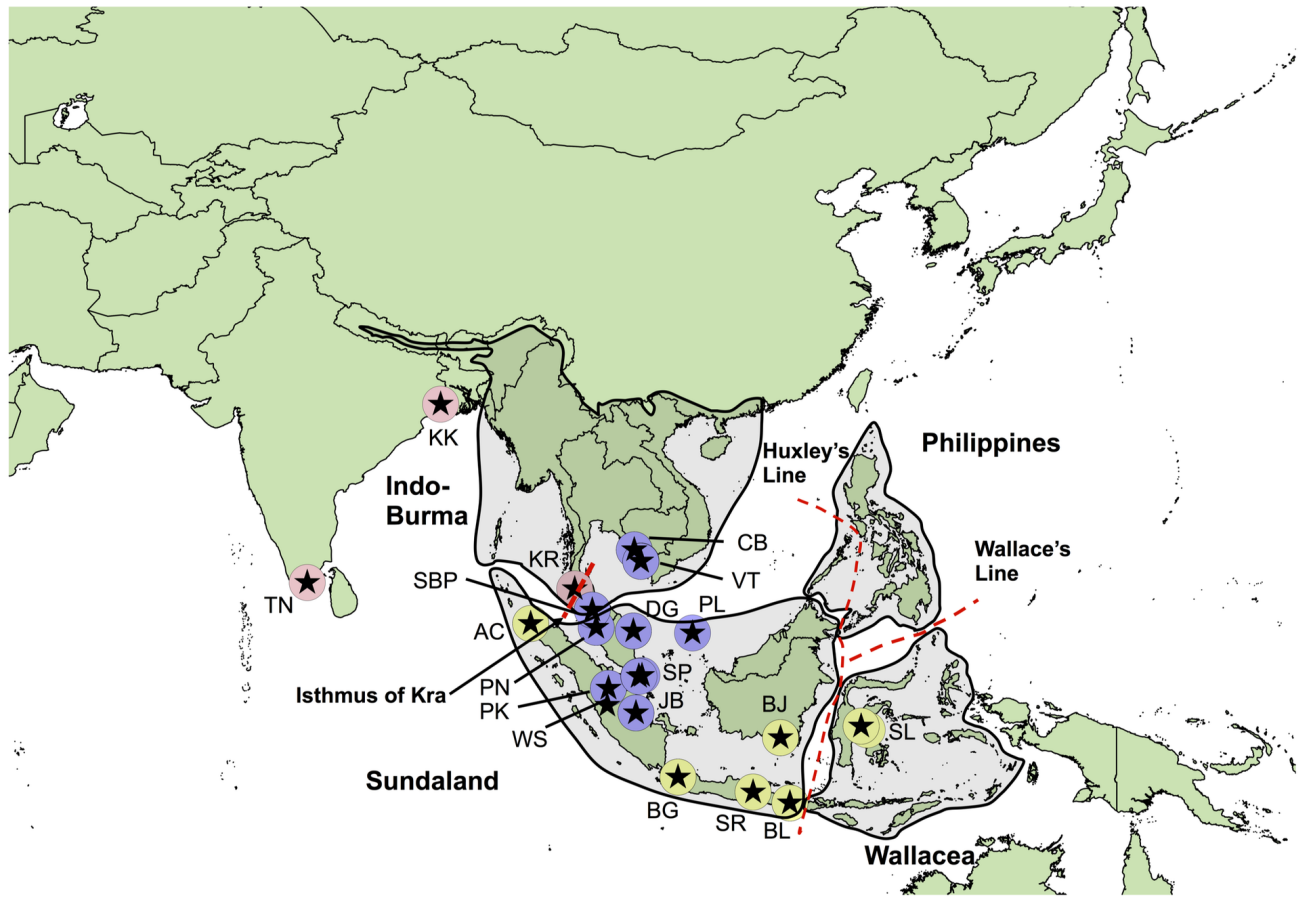
Sampling locations for *Aplocheilus panchax* over 19 areas. Tamil Nadu (TN), Kolkotta (KK), Cambodia (CB), Vietnam (VT), Krabi (KB), Sungai Batu Pahat (SBP), Aceh (AC), Penang (PN), Dungun (DG), Pulau Laut (PL), Singapore (SP), Pekanbaru (PK), West Sumatra (WS), Jambi (JB), Bogor (BG), Surabaya (SR), Banjarmasin (BJ), Bali (BL) and Sulawesi (SL). Points are coloured according to which of the three major mitochondrial clades they correspond to (see ‘Results‘, Fig 2), and stars for locations where nuclear loci were also sampled. Shaded areas indicate the four biodiversity hotspots in Southeast Asia: Sundaland, Wallacea, Philippines and Indo-Burma. Wallace’s Line, Huxley’s modification of Wallace’s Line (based on zoological data; [8]) and the Isthmus of Kra are demonstrated by the red dashed lines.

**Fig 2:**
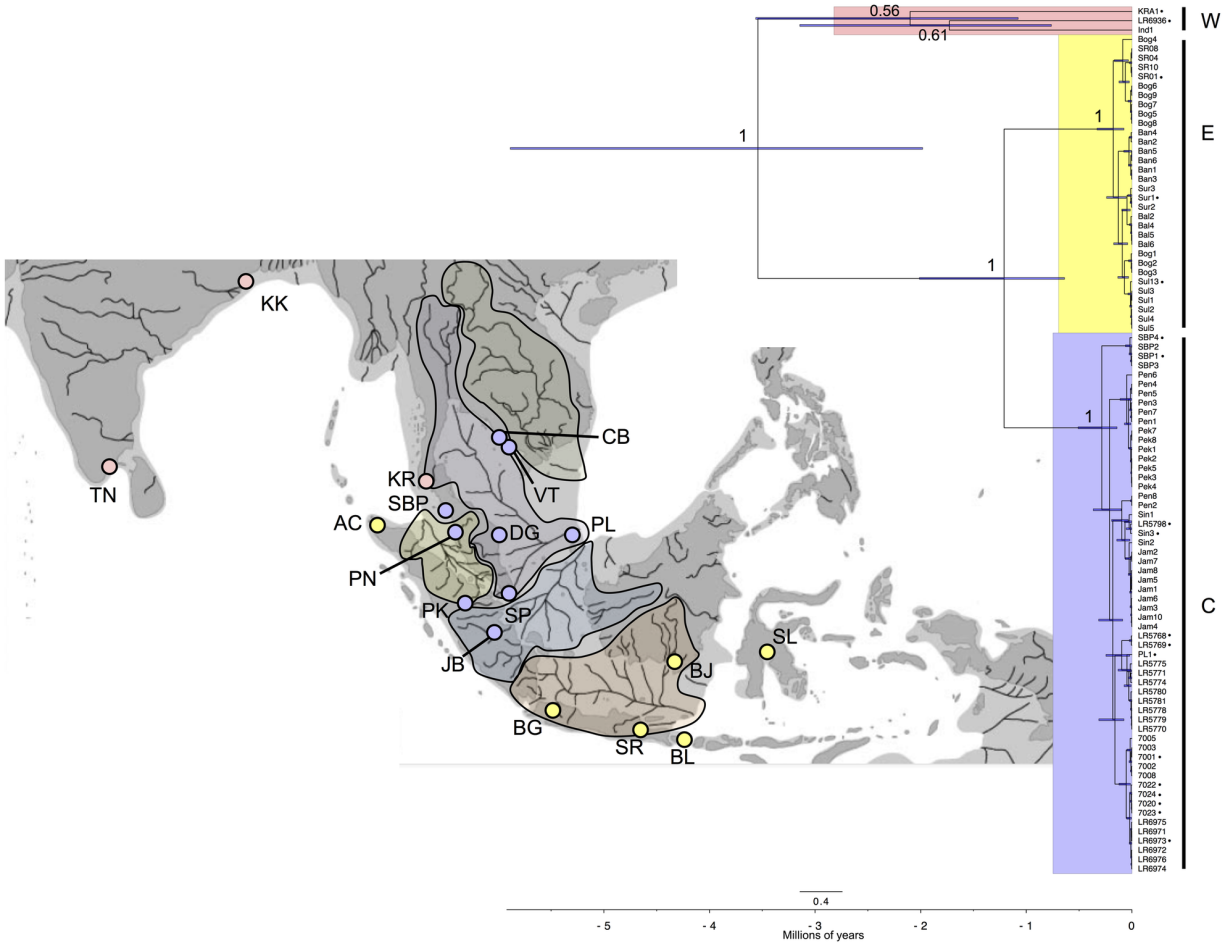
A time calibrated mitochondrial tree of *Aplocheilus panchax*. Bayesian posterior probabilities displayed for each major clade: Western (W), Eastern (E) and Central (C), and node heights showing 95% highest posterior densities. Colours correspond to the three major clades and dark grey shaded areas in SE Asia indicate Pleistocene palaeodrainages (9). Individuals with missing data are still included in the mitochondrial tree (indicated by black circles). See Fig 1 for sampling location abbreviations.

Southeast (SE) Asia comprises only 4% of the World’s terrestrial regions but harbours almost one quarter of its plant and animal species [1]. Four biodiversity hotspots (Sundaland, Indo-Burma, Philippines and Wallacea; Fig 1; [2]), each with their own unique geographic history, have been assigned to this region due to their incredibly high levels of species richness and endemism. This rich biodiversity is attributable to SE Asia’s position on the Asian and Australian biogeographic divide, its history of dramatic sea level changes resulting in repetitive habitat fragmentation, and also being situated within the tropics [3]. It is estimated that 24–63% of the region’s terrestrial species will be threatened with extinction within the next century [4]. However, freshwater biodiversity is experiencing declines at an even higher rate than terrestrial biota as a result of numerous anthropogenic pressures [5]. The repetitive nature of environmental fluctuations during the Plio-Pleistocene not only increased biodiversity, but also shaped contemporary geographic distributions as populations expanded and contracted their ranges in response to sea-level fluctuations [6,7]. Increasing our understanding into how climatic oscillations have changed species distributions in the past could facilitate our predictions of how present-day species will respond to future environmental changes. Here, we demonstrate that climatic oscillations during the Plio-Pleistocene can have a profound effect on the evolutionary history of freshwater fish that are not entirely restricted to freshwaters.

### Sundaland

During the Pleistocene, low sea levels increased connectivity of terrestrial habitats’ across Sundaland, whilst periods of highstands resulted in extensive island archipelagos. Sumatra, Borneo and Java were once connected to continental Asia by a vast landmass, the Sunda Shelf (hereafter referred to as Sundaland), which is now submerged below sea-level [3]. Sundaland was, however, entirely exposed for the majority of the past 2 Myr, as sea levels were below -30m [3]. The exposure of land not only facilitates dispersal for terrestrial species, but also reconnects river systems for dispersal of freshwater species [9,10]. Another land bridge that was frequently exposed lies between the Malay Peninsula and Sumatra, the shallow and narrow nature of the Malacca straits likely facilitated great waves of biotic exchange between these islands [6]. It is thought that this cyclical nature of isolation and reconnection contributes to this region’s high level of species richness and endemism [3]. Contemporary lineages that have persisted through these glacial cycles provide an insight into how species respond to such changes in the environment, whether by relocation [11], adaptation [12], or extinction [13].

Our knowledge into the mechanisms driving biotic evolution in SE Asia has been greatly advanced by combining geological and climatic information with phylogeographic studies on widespread species [14]. Such use of widespread species have enabled the delineation of zoogeographic boundaries, contributing to a better realisation of this region’s remarkable biodiversity [15–17]. Widespread freshwater organisms in particular are ideal for determining the relationship between genealogy and landscape evolution, as they are (mostly) restricted to their two-dimensional habitats, therefore relocation opportunities are limited. For example, Adamson *et al*. [18] used a widespread sedentary freshwater fish species to deduce drainage re-alignments in SE Asia. Phylogeographic signatures indicated that the middle Mekong River Basin may have evolved through an amalgamation of at least three historically independent drainage basins. Sundaland was once drained by numerous vast palaeo-river systems during sea level lowstands (≥ -30m), which may have facilitated the exchange of freshwater organisms between previously, and presently, isolated islands [10]. Glacioeustatic sea level fluctuations and climatic oscillations repeatedly altered drainage connectivity and may have acted as a ‘species pump’ as range fragmentation and vicariance drove population divergence; however, where complete speciation had not yet occurred, genetic admixture may have taken place during ‘reconnection’ events [15,19–21]. The presence or absence of ocean passages had a significant impact on species distributions by limiting species expansions and contractions and ultimately driving evolutionary diversification (e.g. deBruyn *et al*. [21]).

Aplocheiloidei are a suborder of killifish that inhabit both brackish and freshwater. Their global distribution throughout the tropics makes this suborder particularly interesting for biogeographic studies due to conflicting explanations regarding patterns of historical biogeography in the tropics, and their classification as a “secondary division of fresh-water fishes” (i.e. those families that are able to withstand periods of exposure to salinity; [22–27]). Aplocheiloidei consists of three families: 1) Aplocheilidae, endemic to Madagascar, Seychelles and SE Asia; 2) Nothobranchia, endemic to sub-Saharan Africa; and 3) Rivulidae, which are endemic to Middle and South America [27]. A recent phylogeographic study on a widely distributed annual killifish (*Notobranchius orthonotus*) found high genetic differentiation among populations in very close proximity (within a few kilometers) as a result of rivers acting as barriers to gene flow [28]. However, genetic data was not congruent with phenotypic diversification, and it was concluded that *N*. *orthonotus* should be considered a single species with a wide distribution, high morphological variability and strong population structure [29]. In this study, we use multilocus genetic data from the widespread common fresh/brackish non-annual killifish, *Aplocheilus panchax*, to determine whether palaeodrainage connectivity can also shape phylogenetic relationships in organisms that are not entirely restricted to freshwaters. *A*. *panchax* is distributed from Pakistan, throughout SE Asia including the Philippines [30], and is believed to have crossed the Wallace Line naturally into Sulawesi [31,32]. Despite current genetic evidence to indicating a single species [24], its widespread distribution and colour polymorphism may be indicative of a species complex, and/or the presence of extensive levels of cryptic diversity.

Both mitochondrial and nuclear sequences were used in combination with modified palaeodrainage maps [9] to provide a framework by which to determine the role that palaeodrainage connectivity has played in shaping phylogenetic relationships and diversity in fresh/brackish species. We specifically asked whether sea level changes isolated *A*. *panchax* in palaeodrainages, resulting in population fragmentation. In such a scenario, we expected to see evidence of divergence of mitochondrial markers that reflect the distribution of populations within palaeodrainage basins. However, if there has been more recent gene flow between previously isolated populations, then patterns of historical isolation may be lost at nuclear markers.

## Materials and methods

### Ethics statement

Research permits for this study were obtained from the Forest Research Institute Malaysia (FRIM), Vietnam National Museum of Nature, Inland Fisheries Research and Development Institute (IFReDI) in Cambodia, and fieldwork in Peninsular Malaysia and Sarawak were conducted under permits issued by the Economic Planning Unit, Prime Minister’s Department, Malaysia (UPE 40/200/19/2417 and UPE 40/200/19/2534) and the Forest Department Sarawak (NCCD.970.4.4[V]-43). Sample collection was conducted by EN and DW (co-authors of this manuscript) from Indonesian Institute of Sciences (LIPI) and Indonesian Research Institute for Freshwater Aquaculture, respectively. The study was approved by the College of Natural Science Ethics Committee at Bangor University. Fin clips were taken where possible to avoid sacrificing the animal. Any fish that were euthansied was done so according to Bangor Ethics Committee guidelines (using MS-222 Tricaine methanesulfonate).

### Sampling

A total of 94 individuals of *A*. *panchax*, representing 22 locations, were examined (S1 Table; see Fig 1 for geographic sampling of mitochondrial and nuclear loci). Small sample sizes were included to maximise geographic coverage. The majority of samples were collected by the authors and preserved in 95% ethanol, whilst Indian samples (IND1 and LR6936) were sourced from the ornamental fish trade. The geographic co-ordinates of each location were determined by GPS, except for the Indian samples (IND1 and LR6936), which were determined from GoogleEarth (GE), using approximate source locations. DNA sequence variation was examined at two mitochondrial markers (cytochrome oxidase I (COI) and control region (CR)), and three anonymous nuclear loci (AP44, AP50 and AP70; [33]).

### Sequencing and alignments

Whole genomic DNA (see S1 Table for sample sizes) was extracted from fin clippings using the high-salt DNA extraction protocol [34]. CR and COI were PCR amplified and sequenced following the methodology described in deBruyn *et al*. [21]. In addition, sequence data for three anonymous nuclear loci (AP44, AP50 and AP70) were generated for a subset of individuals using the primers and PCR conditions described in deBruyn *et al*. [33]. Not all loci amplified reliably across samples (S1 Table), but were included to maximise geographic sampling. All PCR samples were accompanied by negative controls. PCR products were purified using a Qiagen purification kit and sequenced by Macrogen Inc. (http://www.macrogen.com). Sequence chromatograms were aligned using the neighbour-joining (NJ) default option in muscle [35]. Minor adjustments were then made by eye to manually remove any false homologies. Protein-coding genes were translated to check for unexpected stop codons or frameshift mutations using the software mega v5.1 [36]. A blast search of all three anonymous nuclear markers revealed no links to any known functional elements.

### Mitochondrial phylogeny

beast v1.7.4 [37] was used to jointly estimate the mitochondrial phylogeny and divergence timings using a Bayesian approach. To calibrate the mitochondrial tree, we applied a normal prior on the CR substitution rate with mean 0.017 mutations/site/Myr and standard deviation 0.0025 mutations/site/Myr. This prior provided upper and lower 95% confidence intervals of 0.022 and 0.012 mutations/site/Myr respectively, which encompasses published CR substitution rates of various families of fishes [38]. The COI substitution rate was estimated relative to CR. *A*. *panchax* population size may have fluctuated through time, and so a coalescent tree prior with exponential population growth rate was specified to accommodate such changes. Appropriate substitution models were chosen for CR and COI using mega v5.1 [36] under the Bayesian Information Criterion (BIC: [39]). Preliminary beast runs using lognormal relaxed clock models failed to reject a constant evolutionary rate. A strict clock model was therefore specified for each gene in the final analysis. The analysis consisted of two independent runs of 10^7^ generations, each sampling the Markov Chain Monte Carlo (MCMC) chain every 1000 generations. The first 10% of each run was discarded as burn in. Tracer v1.4 [40] was used to verify convergence within and between the two runs, and to check that burn-in was sufficient. The runs were combined and a maximum clade credibility (MCC) time-calibrated tree was then selected from the posterior sample of trees using TreeAnnotator, and annotated with posterior clade probabilities and 95% confidence intervals (CI) for node ages. Following the identification of distinct mitochondrial clades in *A*. *panchax*, levels of genetic divergence were estimated under the Kimura 2-parameter model (K2P; [41]) in mega. The K2P method was used to enable the standardisation of comparisons amongst studies, as recommended by the Consortium for the Barcoding of Life (http://www.barcoding.si.edu/protocols.html).

### Phylogeographic diffusion analyses

The geographic location of the most recent common ancestors of *A*. *panchax* clades were estimated from the geo-referenced mitochondrial data using a continuous phylogeographic diffusion model in beast. We selected the mitochondrial dataset as a basis for phylogeographic inference as this provided greater phylogenetic signal than nuclear markers, and the alternative of concatenating multiple markers is known to potentially mislead phylogenetic inference [42]. A Cauchy relaxed random walk (RRW) diffusion model was used, which accommodates variation in geographic dispersal over time and across different lineages of the phylogeny [43]. Examination of preliminary runs showed that posterior distributions of parameters describing variation in dispersal rate excluded zero, therefore rejecting a constant rate of dispersal, and justifying the use of the RRW model. Evolutionary rate calibration and tree prior were as described for the mitochondrial phylogeny. Duplicate localities can confound the RRW diffusion model; therefore a biologically realistic random jitter was applied to duplicate locations, which randomly selected new localities from a 0.02° x 0.02° geographic window centred on the original locality. Two independent runs of 1 x 10^8^ generations were conducted, sampling the MCMC chain every 5,000 generations with the first 10% removed as burn-in. A MCC tree was generated (as previously described) using a single run, and the program spread [44] was used to generate a KML file for visualisation in Google Earth.

### Demographic history and genetic diversity

To explore the effect that sea level fluctuations had on the current distribution of *A*. *panchax*, a mismatch distribution analyses was performed using concatenated mitochondrial sequences from two of three focal mitochondrial clades (see ‘Results‘), as sample size was too small in the Western clade (n = 3). Using a mismatch distribution, genetic signatures of population expansion can be determined via the distribution of pairwise nucleotide differences [45]. Unimodal and smooth wave-like distributions suggests an expanding population, whereas a multimodal distribution suggests a stable population. The sum of squared deviation (SDD) and Harpending’s raggedness index (RI) was calculated for the observed mismatch distributions and compared with data simulated from the sudden expansion model. Further tests for population expansion was obtained using Tajima’s *D* [46] and Fu’s *Fs* [47] neutrality tests. Any departure from the assumption of neutrality indicates the occurrence of non-neutral processes, such as gene flow, changes in population size or selection. Negative values are indicative of a recent population expansion, with significant p-values rejecting the null hypothesis of a constant population size. Haplotype diversity and nucleotide diversity was also estimated for the two mitochondrial clades. All tests were conducted in Arlequin v3.5.2.2 [48].

### Nuclear DNA analysis

phase v2.1.1 [49,50] was used to infer individual haplotype/allelic sequences from diploid nuclear sequences. The program seqphase (www.mnhn.fr/jfflot/seqphase: [51]) was used for phase input and output file conversion. phase was run twice with different starting seeds, with each run consisting of 100 iterations preceded by a burn-in phase of 100 iterations. Substitution models were selected for each anonymous nuclear marker under the BIC in mega v5.1. Individual gene trees were inferred for each nuclear locus using beast. A constant evolutionary rate was rejected for AP44 and AP50 but not for AP70, therefore relaxed lognormal clock models were used for the former two markers and a strict clock model for the latter. The details of the MCMC chain, Tracer analysis and tree annotation were as described previously. Median joining (MJ) nuclear haplotype networks were produced using the program Network (www.fluxus-engineering.com; [52]), and checked for congruence with mitochondrial clades and geography.

In addition, combined nuclear genetic variation was also examined using a distance-based approach. Initially, allele distance matrices were generated for each nuclear locus under the K2P model [41] in mega. The program pofad v.1.03 [53] was then used to convert these allelic distances to matrices of pair-wise genetic distances between individuals, to rescale distances so all loci were equally weighted, and then to generate a final matrix of standardised multilocus genetic distance between individuals, calculated as the mean rescaled distance across all loci. Some individuals had sequences only for two nuclear markers, so overall genetic distance for these individuals was calculated as the mean of those loci for which data were available. Standardised multilocus distances were then used to cluster individuals based on genetic similarity using principal co-ordinates analysis (PCoA), which ordinates individuals in multidimensional space based on pairwise genetic similarity, and then calculates the axes (principal co-ordinates) accounting for the most variation within this space.

We also performed Mantel tests [54] to test alternative causal hypotheses likely explaining the observed geographic patterns of nuclear genetic variation. Specifically, we assessed the relative importance of: 1) historical isolation (as predicted by patterns of mtDNA variation); 2) recent gene flow (the magnitude of which we assume to correlate with geographic proximity = isolation-by-distance); and 3) palaeodrainage assignments, in explaining the standardised multilocus nuclear genetic distance matrix generated in pofad. Two categorical matrices were constructed with individuals from the same mitochondrial clade coded as 1, and those from different mitochondrial clades coded as 2, and the same coding method was performed in the second categorical matrix to test for palaeodrainage assignments. To test the effect of geographic distance on multilocus nuclear distance (isolation-by-distance), we generated a matrix of pairwise geographic distances from geospatial co-ordinates using the Geographic Distance Matrix Generator v. 1.2.3 [55]. Individuals sampled from the same locality were arbitrarily assigned a pairwise distance of one metre, in order to remove zero values from the geographic distance matrix. Partial Mantel tests were performed to test the effect of mitochondrial clade affinity, geographic proximity and palaeodrainage assignment on nuclear genetic distance, whilst controlling for the opposing variables. Calculations were performed using the Isolation by Distance Web Service with 30,000 matrix randomisations [56].

## Results

### Sequence data

A total of 988 base pairs (bp) of mitochondrial sequence data (621 bp COI, 367 bp CR) and 1102 bp of nuclear sequence data (548 bp Ap44, 224 bp AP50, 330 bp Ap70, see Table 1) were aligned. Substitution models identified by model testing for both mitochondrial and nuclear markers are shown in Table 1. Conversion of the COI nucleotide sequences into amino acid sequence indicated that nearly all polymorphisms were synonymous substitutions. Only one amino acid change was inferred, from an Indian sample (‘Ind 1’). Moreover, no stop codons were identified, consistent with a lack of pseudogenes in the COI dataset.

**Table 1:** Loci information and selected evolutionary models.

Locus	BIC	bp	n^a^	No. of variable sites
COI	HKY+G	621	78	51
CR	HKY+G	367	89	47
AP44	TN93+G	548	40 (80)	37
AP50	HKY	224	44 (84)	22
AP70	HKY+G	330	40 (80)	26

Model testing results (BIC); bp, number of base-pairs; n, sample size.

^a^ Sample size after phase analyses for nuclear sequences are shown in parentheses.

### Mitochondrial phylogeny

The basal divergence of *A*. *panchax*, representing the divergence of Indian (Ind1 and LR6936) and Krabi (Kra1) mitochondrial lineages, from the remaining samples included here, was estimated at ~3.52 Ma (Fig 2). The second major split in the phylogeny, between the Indian and Krabi lineages, occurred ~2.2 Ma, with the third major split occurring ~1.2 Ma, during the Mid-Pleistocene, resulting in two additional major clades (Central and Eastern) comprising the majority of our samples (central Sundaland and eastern Sundaland/Sulawesi, respectively). This split was followed by substantial within clade diversification within the last ~150 kyr (Fig 2). An unexpected relationship was the phylogenetic affinity of samples from Aceh, Sumatra, which grouped with the Eastern clade instead of the Central clade as would be expected based solely upon geographic proximity (Fig 2). The mitochondrial tree (Fig 2) showed little relationship between the distribution of lineages of *A*. *panchax* and palaeodrainage basin distributions (S1 Fig).

### Phylogeographic diffusion analyses

The median ancestral locations and 80% Highest Posterior Density (HPD) contours are shown in S2 Fig. Based on our sampling, the expansion of extant *A*. *panchax* mitochondrial lineages initially progressed relatively slowly within the Thai-Malay Peninsula, starting some 3.4 Ma, eventually arriving in East Java around 173 ka (S2 Fig). A simultaneous dispersal event out of Peninsula Malaysia and West Java occurred soon after (115 ka), which was then followed by a rapid radiation across SE Asia including possible dispersal from West Java (Bogor) to Aceh, and colonisation of Sulawesi from Surabaya (East Java) ~27 ka. Pulau Laut (Natuna) was also colonised during this time period from Malaysia; both mainland and island localities fall within the Siam palaeodrainage system that connected these two areas at this time [9,21]. As sampling across SE Asia was not exhaustive, at this stage we cannot rule out alternative colonisation pathways, or the wholescale replacement of pre-existing lineages. It was evident, however, that the Pleistocene radiation of extant mitochondrial lineages across SE Asia occurred in a predominantly west-east direction.

### Demographic history and genetic diversity

**Fig 3:**
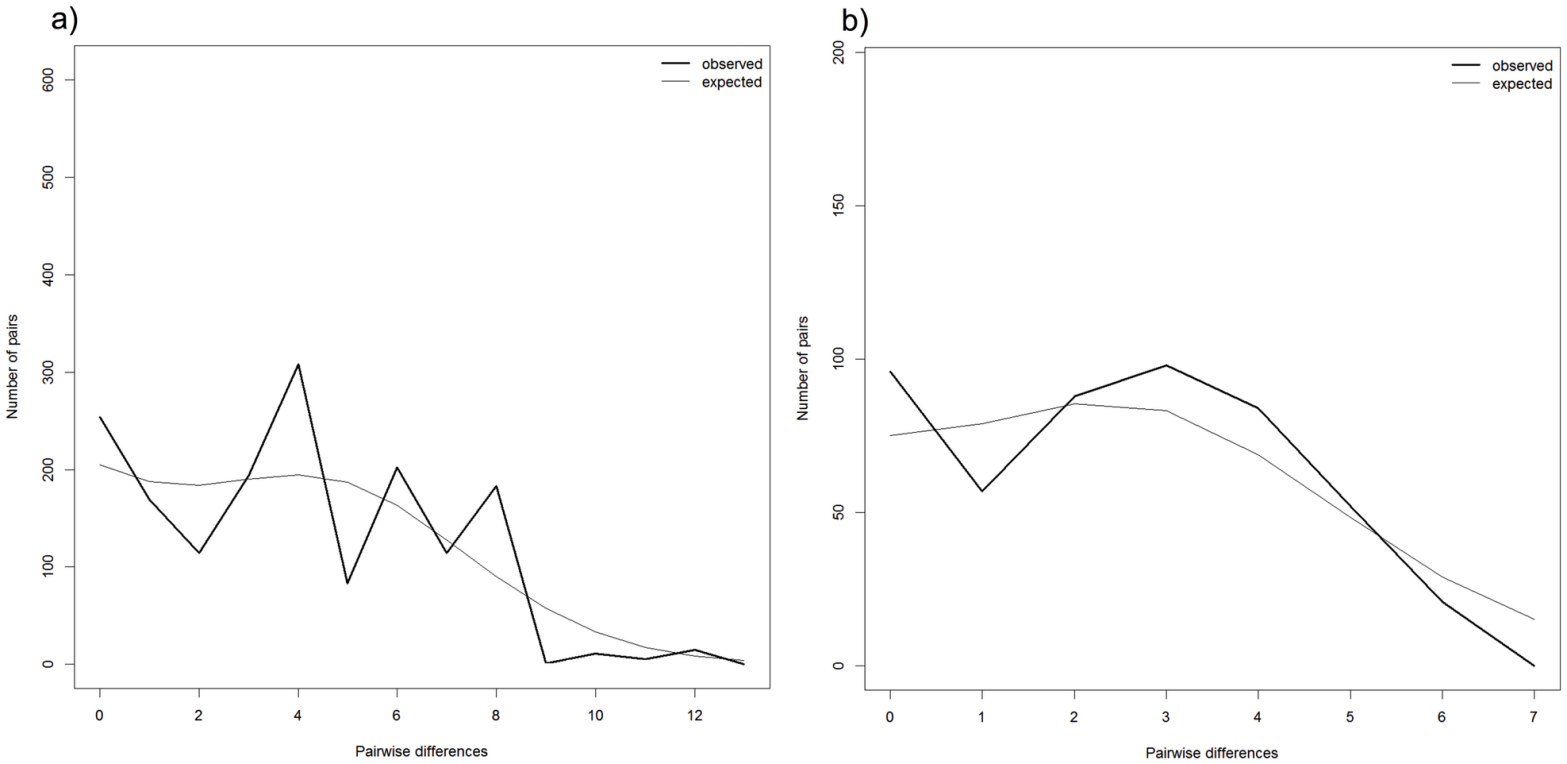
Mismatch distributions for *Aplocheilus panchax*. Mismatch distribution curves for the Central (a) and Eastern (b) mitochondrial clades showing the expected (thin line) and the observed (bold line) values under the population expansion model.

Observed mismatch distributions for the Central clade was more representative of a multimodal distribution, unlike the Eastern clade which had a more unimodal distribution (Fig 3). The data was unable to reject the null hypothesis of a constant population size (P > 0.05) for both clades (Table 2). Fu’s *Fs* was negative for both clades indicating population expansion, but only significant for the Central clade (P < 0.01). Tajima’s *D* was also negative for the Central clade but not for the Eastern clade, neither of which were significant.

**Table 2:** Results of genetic diversity and demographic analyses on the Central and Eastern mitochondrial clades.

Clade	n	bp	vs	H	Hd	π	*D*	*Fs*	SSD	RI
Central	58	988	27	21	0.9159 (0.02)	0.004 (0.002)	-0.114	**-7.324**^a^	0.017	0.051
Eastern	32	988	8	10	0.8569 (0.04)	0.003 (0.002)	0.901	-1.985	0.006	0.021

n, number of individuals; bp, base pairs; vs, variable sites; H, number of haplotypes; Hd, haplotype diversity; π, nucleotide diversity.

^a^ Significant values are indicated in bold (P<0.05).

### Nuclear allele networks

**Fig 4:**
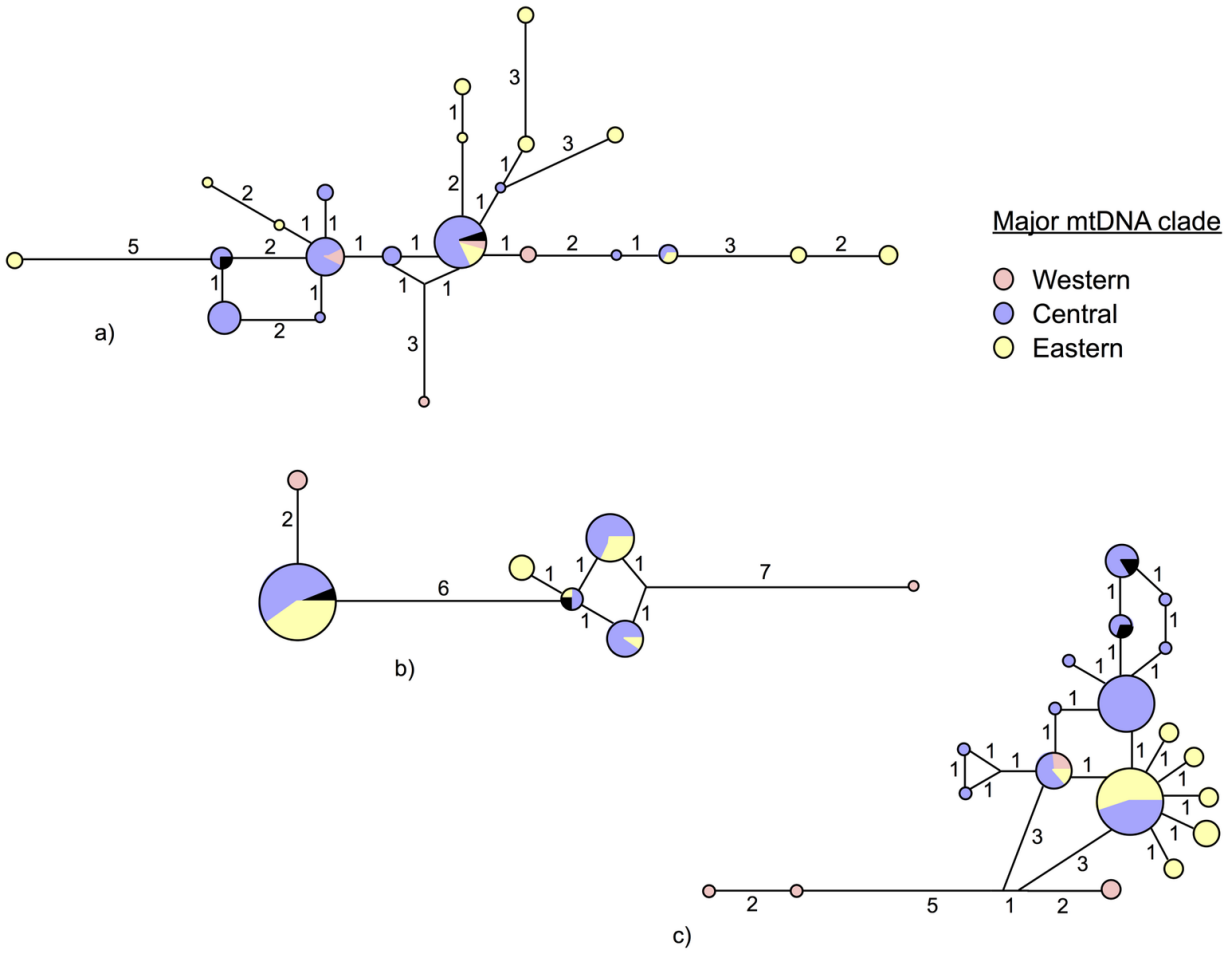
Median-joining nuclear allele networks for *Aplocheilus panchax*. Colours correspond to mitochondrial clades (see Fig 2). Node size is proportional to allele frequency and numbers indicate the number of mutations. Black sections are those alleles not included in the mitochondrial analyses (a single sample from west Sumatra). a) AP44, b) AP50 and c) AP70.

The MJ nuclear allele networks for all three anonymous nuclear markers do not show any obvious relationship with either geographic distribution (S3 Fig) or mitochondrial clade affinity (Fig 4). However, results from the partial Mantel Test of nuclear genetic distances indicate otherwise (see below). Despite this discordance, both AP50 and AP70 displayed large numbers of mutations, reflecting the deep divergence of the Indian samples (Fig 4b and 4c).

### Mantel tests

The results of partial Mantel tests are shown in Table 3. These results revealed nuclear genetic distances to be significantly associated with geographic proximity, mitochondrial clade affinity and palaeodrainage assignments. Taking into account the effect of mitochondrial clade affinity and palaeodrainage assignments, significant associations remained with geographic proximity. However, with the inclusion of geographic proximity, nuclear genetic distance was no longer found to be significantly associated with neither palaeodrainage basins, nor mitochondrial clade affinity. Overall, our results indicate that recent gene flow as opposed to historical isolation has been the key factor determining patterns of nuclear genetic variation in *A*. *panchax*.

**Table 3:** Results of Mantel tests of matrix correlation between standardised nuclear genetic distance, geographic distance and mitochondrial clade affinity. *Z* = Mantel test statistic. *r* = correlation coefficient. partial *r* = partial correlation coefficient, controlling for the effect of the opposing variable. Significant values (*P* <0.05) are shown in bold.

Test group	*Z*	*r*	Partial *r*
mitochondrial clade	309	**0.2237**	0.044
geographic distance	317555	**0.336**	**0.2608**
palaeodrainage basin	360	**0.0883**	-0.0742
geographic distance	317555	**0.336**	**0.333**

## Discussion

The findings revealed three major mitochondrial clades across the distribution of *A*. *panchax*. Although the CI’s surrounding the divergence timing of mitochondrial lineages are relatively large, the time-calibrated mitochondrial tree suggests that initial splitting of the major lineages, distributed to the north and south of the Isthmus of Kra, respectively, coincided with the Pliocene (3.5 Ma). Krabi is situated <400km away from the Central lineage, yet it shares a mitochondrial haplotype with geographically distant lineages from India (Western clade), suggesting a vicariance scenario (Fig 2). A study of giant freshwater prawns (*Macrobrachium rosenbergii*; [57]) also revealed a similar pattern of subdivision of populations north and south of the Isthmus of Kra in freshwater-dependent fauna.

Major divergence events coincided initially with the early Pleistocene (~2 Ma), a pattern similar to that identified for some Northern Hemisphere fishes (e.g. April *et al*. [20]), and progressed through the Pleistocene. Further lineage splitting occurred some 1.2 Ma, subdividing samples from Sundaland into two monophyletic mitochondrial clades, a Central and an Eastern clade (Fig 2). Surprisingly, samples from Aceh, Sumatra, grouped with the Eastern clade, which is unexpected based on geographic proximity (Fig 2). Human introduction and long-distance dispersal seem unlikely scenarios given the large geographic distances involved and that colonisation supposedly occurred ~20ka. Stepping stone dispersal via estuaries along the coast, as well as microvicariant events during Pleistocene transgressive and regressive episodes, are more likely scenarios that require further examination. The isolation and divergence of a once widespread population via marine transgressions has been documented before in the New World family of Killifish, Rivulidae [58,59].

Based on phylogeographic diffusion analyses of mitochondrial data, a slow southward expansion of ancestral *A*. *panchax* took place along the Thai-Malay Peninsula, with colonisation of Java occurring only around ~170 ka (S2 Fig). Indeed, geological evidence indicate that east Java was submerged prior to this time [60]. This colonisation was followed by a simultaneous expansion in the Malaysian and Javan regions, extending into Bali, Borneo, and Sulawesi starting ~120 ka, coinciding with the start of the last glacial cycle, when sea levels fell from +6m at 120 ka, to -124m and -130m during the last glacial maximum, 19–26 ka [3]. Based on diffusion modelling, most populations included appear to have been founded during this last glacial cycle, including Vietnam, central and south-eastern Sumatra, southern Borneo, east Java, Bali and Sulawesi, though replacement of pre-existing lineages cannot be excluded. Based on our data, Sulawesi appears to have been colonised from Surabaya (east Java), which has been separated by a vast expanse of ocean since the opening of the Makassar Strait ~45 Ma which separated west Sulawesi from Sundaland [60,61]. Divergence timings indicate that *A*. *panchax* may have only crossed the Wallace Line into Sulawesi ~27ka. Existing evidence (e.g., Klaus *et al*. [15]) suggests that it is more likely that the arrival of *A*. *panchax* in Sulawesi occured from east Borneo, likely via a freshwater surge from the large river mouth of the Mahakam River. The most dominant ocean current in the Makassar Strait is from north to south, making it possible for rafts and freshets—carrying both terrestrial and freshwater species—to arrive at the narrows of the Makassar Strait in Sulawesi; whilst the lack of major rivers in Sulawesi comparable to the size and position of the Mahakam River has likely prevented the flow of return ‘traffic’ (Voris, H. 2015, personal communication). Additionally, Lindsey and McPhail [62] noted that temporary freshwater or brackish-water bridges can form during periods of high run-off, facilitating the dispersal of freshwater organisms across saltwater gaps. An example of this dispersal mechanism comes from a study of Mesoamerican freshwater fishes, which suggests that secondary freshwater fishes (including Cichlidae and Aplocheiloids) colonised Mesoamerica before the uplift of the Isthmus of Panama as a result of salinity tolerance coupled with large flooding events [63,64]. It is therefore important to consider these mechanisms of dispersal in biogeographic studies.

The lowering of sea levels would have re-connected palaeodrainages, presumably facilitating *A*. *panchax* dispersal throughout Sundaland and/or possibly via stepping-stone dispersal between adjacent palaeo-estuaries along the coast, a scenario that is consistent with partial Mantel test results (Table 3). Surprisingly, little congruence was identified between the distribution of lineages of *A*. *panchax* and the currently recognised geographic distribution of palaeodrainage basins (S1 Fig), suggesting that estuarine ‘hopping’ or river capture events also likely facilitated their dispersal. However, Pulau Laut’s close genetic relationship to samples from the east coast of Peninsula Malaysia does offer limited support for palaeodrainage connectivity.

Nuclear data (Fig 4), in contrast to the phylogeographic structure evident from mitochondrial data, shows a lack of phylogeographic structure across SE Asia. Such mito-nuclear discordance may have arisen by two processes: either the retention of shared ancestral genetic polymorphism in nuclear loci among geographically isolated populations (incomplete lineage sorting) due to the four-fold larger effective population size of nuclear loci in comparison to mtDNA, or alternatively via secondary contact and male-biased admixture (e.g. Croft *et al*. [65]) between previously isolated populations, which would have retained the signature of past isolation in mtDNA during population reintegration. These two alternative causal factors may be distinguishable by examining the broad scale patterns of nuclear variation. Incomplete lineage sorting, being a random process, is unlikely to result in an association between genetic similarity and geographic distance. However, recent gene flow is likely to produce a significant isolation by distance pattern in this dispersal-limited species.

Initial Mantel tests showed a significant association of nuclear genetic distance with geographic distance, mitochondrial clade and palaeodrainage assignment; however, when we controlled for the opposing variable, geographic distance remained significant, but not mitochondrial clade nor palaeodrainage affinity. Recent gene flow thus appears to be the main factor shaping patterns of nuclear variation, rather than historical isolation. The alternative hypothesis of reproductive isolation with incomplete lineage sorting of nuclear markers would not predict a significant isolation-by-distance relationship. Here, the combined analyses of nuclear and mtDNA markers suggests that: firstly, mtDNA clades are not cryptic species/reproductively isolated units, though further investigation is required for the highly divergent Indian lineages; secondly, *A*. *panchax* has undergone historical isolation, resulting in distinct mtDNA clades, but these clades have intermixed on secondary contact, resulting in an isolation-by-distance pattern for nuclear DNA; and finally, the discrepancy between mitochondrial and nuclear DNA suggests that gene flow may be male-mediated. Based on the current sampling of individuals and genetic loci, estimating the magnitude of this geneflow, and directly testing the male-biased geneflow hypothesis, is beyond the scope of this study, but would be a valuable direction for future research. Overall, emergent patterns disclosed here support a hypothesis of vicariant isolation, followed by range expansion and secondary contact across Sundaland during periods of low sea levels and maximum freshwater connectivity, primarily during the last glacial cycle [66].

## Conclusions

*Aploceheilus panchax*’s wide geographic distribution has not only shed further light on the roles of various biogeographic barriers in shaping biodiversity across SE Asia, but has also contributed to our understanding of killifish biogeography, a highly controversial topic stemming from Murphy’s [67] classification of Aplocheiloids as being secondary freshwater fish. This study demonstrates a combination of vicariance and dispersal events, associated with sea level fluctuations, have contributed to the current distribution of *A*. *panchax* in SE Asia. Although the use of such a widespread brackish species may be limited in delineating biogeographic boundaries, they can instead lead us towards a deeper understanding of how climatic oscillations can facilitate the dispersal of organisms across supposedly major barriers to gene flow. To the best of our knowledge, no study has yet documented evidence for a freshwater flux from east Borneo across the Wallace Line into Sulawesi. This possible mechanism of dispersal could likely have resulted in numerous terrestrial and freshwater species occurrences in the ‘anomalous’ island of Sulawesi.

Considerable lack of knowledge of biodiversity in the tropics currently limits our ability to quantify the true extent of freshwater biodiversity, as well as limiting our ability to alleviate potential threats [5]. Our study adds to the mounting body of evidence [15,18,21,68–73] indicating that freshwater diversity within SE Asia is currently underestimated, and is in urgent need of further research and conservation effort.

## Supporting information

S1 Table Sample information. Details of samples, sequences and corresponding GenBank Accession numbers for *Aplocheilus panchax*. Co-ords indicate whether sample locations were determined by GPS or GoogleEarth (GE). The exact locality of L6936 is unknown but originated in Kalkotta.
(PDF)

S1 Fig Mitochondrial palaeodrainage map. A time calibrated mitochondrial tree with 95% highest posterior densities. Southeast Asia’s palaeodrainage basins are coloured, as well as corresponding mitochondrial samples. Those samples that do not fall within a drainage basin, possibly as a result of stepping-stone dispersal, are left uncoloured. Western (W), Eastern (E) and Central (C) mitochondrial clades are also indicated.
(TIF)

S2 Fig Phylogeographic diffusion model. Map of SE Asia visualising dispersal timings of *Aplocheilus panchax* using molecular dating from the time calibrated mitochondrial tree (see Fig 2 in main text): a) 3.39Ma, b) 173,486ka, c) 114,987ka and d) 27,237ka. Black shaded areas indicate the confidence surrounding the ancestral locations, whilst blue shaded areas show 80% HPD.
(PDF)

S3 Fig Median-joining nuclear allele networks based on geographic distribution. Median-joining nuclear allele networks for *Aplocheilus panchax* for three anonymous nuclear markers: a) AP44, b) AP50, and c) AP70. Colours correspond to geographic location, node size is proportional to allele frequency and numbers indicate the number of mutations.
(PDF)
