# Supplementary material for: Plio-Pleistocene phylogeography of the Southeast Asian Blue Panchax killifish, Aplocheilus panchax
Source: PLoS One. 2017 Jul 25;12(7):e0179557. doi: 10.1371/journal.pone.0179557 (PMC5526567; doi:10.1371/journal.pone.0179557)
Supplement: S1 Table — Details of samples, sequences and corresponding GenBank Accession numbers for Aplocheilus panchax. Co-ords indicate whether sample locations were determined by GPS or GoogleEarth (GE). The exact locality of L6936 is unknown but originated in Kalkotta. (PDF) [file pone.0179557.s001.pdf]

S1 Table. Details of samples, sequences and corresponding GenBank Accession numbers for *Aplocheilus panchax*. Co-ords indicate whether sample locations were determined by GPS or GoogleEarth (GE). The exact locality of L6936 is unknown but originated in Kalkotta.

Sample	Country	Locality	Latitude	Longitude	Co-ords	CR	CO1	AP44	AP50	AP70
**Pen1**	Malaysia	Penang	5.1643167	100.4814	GPS	GQ377753	KJ957587	-	-	-
**Pen2**	Malaysia	Penang	5.1643167	100.4814	GPS	GQ377753	KJ957588	-	-	-
**Pen3**	Malaysia	Penang	5.1643167	100.4814	GPS	GQ377753	KJ957610	KJ957303	-	KJ957410
**Pen4**	Malaysia	Penang	5.1643167	100.4814	GPS	GQ377753	KJ957589	-	-	-
**Pen5**	Malaysia	Penang	5.1643167	100.4814	GPS	GQ377753	KJ957590	-	-	-
**Pen6**	Malaysia	Penang	5.1643167	100.4814	GPS	GQ377753	KJ957611	KJ957304	KJ957370	KJ957411
**Pen7**	Malaysia	Penang	5.1643167	100.4814	GPS	GQ377753	KJ957612	KJ957305	KJ957371	KJ957412
**Pen8**	Malaysia	Penang	5.1643167	100.4814	GPS	GQ377753	KJ957613	-	-	-
**Sur1**	Indonesia	Near Surabaya	-7.6599667	112.6979667	GPS	GQ377753	-	-	-	-
**Sur2**	Indonesia	Near Surabaya	-7.6599667	112.6979667	GPS	GQ377753	KJ957554	-	KJ957384	KJ957423
**Sur3**	Indonesia	Near Surabaya	-7.6599667	112.6979667	GPS	GQ377753	KJ957555	-	KJ957385	-
**Bog1**	Indonesia	Bogor	-6.4962	106.8516833	GPS	GQ377753	KJ957546	KJ957284	KJ957348	KJ957392
**Bog2**	Indonesia	Bogor	-6.4962	106.8516833	GPS	GQ377753	KJ957547	-	-	-
**Bog3**	Indonesia	Bogor	-6.4962	106.8516833	GPS	GQ377753	KJ957548	-	-	-
**Bog4**	Indonesia	Bogor	-6.4962	106.8516833	GPS	GQ377753	KJ957549	-	-	-
**Bog5**	Indonesia	Bogor	-6.4962	106.8516833	GPS	GQ377753	KJ957607	KJ957296	KJ957362	-
**Bog6**	Indonesia	Bogor	-6.4962	106.8516833	GPS	GQ377753	KJ957550	KJ957297	KJ957363	KJ957404
**Bog7**	Indonesia	Bogor	-6.4962	106.8516833	GPS	GQ377753	KJ957551	-	-	-
**Bog8**	Indonesia	Bogor	-6.4962	106.8516833	GPS	GQ377753	KJ957552	-	-	-
**Bog9**	Indonesia	Bogor	-6.4962	106.8516833	GPS	GQ377753	KJ957553	-	-	-
**Ban1**	Indonesia	Banjarmasin	-3.4266167	114.83245	GPS	GQ377753	KJ957540	KJ957283	KJ957347	KJ957391
**Ban2**	Indonesia	Banjarmasin	-3.4266167	114.83245	GPS	GQ377753	KJ957541	-	-	-
**Ban3**	Indonesia	Banjarmasin	-3.4266167	114.83245	GPS	GQ377753	KJ957542	-	-	-

**Ban4**	Indonesia	Banjarmasin	-3.4266167	114.83245	GPS	GQ377753	KJ957543	-	-	-
**Ban5**	Indonesia	Banjarmasin	-3.4266167	114.83245	GPS	GQ377753	KJ957544	-	-	-
**Ban6**	Indonesia	Banjarmasin	-3.4266167	114.83245	GPS	GQ377753	KJ957545	-	-	-
**PL1**	Indonesia	Pulau Laut, Natuna	4.7268	107.982	GPS	GQ377753	-	KJ957306	KJ957372	-
**Sin1**	Singapore	Tekong Res.	1.415	104.045	GPS	GQ377753	KJ957616	KJ957310	KJ957376	-
**Sin2**	Singapore	Rifle Range	1.3569444	103.7916667	GPS	GQ377753	KJ957617	KJ957311	KJ957377	KJ957416
**Sin3**	Singapore	Sime Rd	1.3427778	103.8130556	GPS	GQ377753	-	KJ957312	KJ957378	KJ957417
**Kra1**	Thailand	Krabi, Sa Keao	8.1684	98.8091667	GPS	GQ377753	-	KJ957300	KJ957367	KJ957407
**WS1**	Indonesia	West Sumatra	-0.905	101.3458333	GPS	-	-	KJ957318	KJ957386	KJ957424
**IND1**	India	Tamil Nadu	8.6554889	77.9811111	GE	GQ377753	KJ957593	KJ957285	KJ957349	KJ957393
**Jam1**	Indonesia	Jambi	-1.49655	103.5772167	GPS	GQ377753	KJ957565	KJ957298	KJ957364	-
**Jam2**	Indonesia	Jambi	-1.49655	103.5772167	GPS	GQ377753	KJ957566	KJ957299	KJ957365	KJ957405
**Jam3**	Indonesia	Jambi	-1.49655	103.5772167	GPS	GQ377753	KJ957567	-	KJ957366	KJ957406
**Jam4**	Indonesia	Jambi	-1.49655	103.5772167	GPS	GQ377753	KJ957568	-	-	-
**Jam5**	Indonesia	Jambi	-1.49655	103.5772167	GPS	GQ377753	KJ957569	-	-	-
**Jam6**	Indonesia	Jambi	-1.49655	103.5772167	GPS	GQ377753	KJ957570	-	-	-
**Jam7**	Indonesia	Jambi	-1.49655	103.5772167	GPS	GQ377753	KJ957571	-	-	-
**Jam8**	Indonesia	Jambi	-1.49655	103.5772167	GPS	GQ377753	KJ957572	-	-	-
**Jam10**	Indonesia	Jambi	-1.49655	103.5772167	GPS	GQ377753	KJ957573	-	-	-
**Pek1**	Indonesia	Pekanbaru	0.4220333	101.4367167	GPS	GQ377753	KJ957582	-	-	-
**Pek2**	Indonesia	Pekanbaru	0.4220333	101.4367167	GPS	GQ377753	KJ957583	-	-	-
**Pek3**	Indonesia	Pekanbaru	0.4220333	101.4367167	GPS	GQ377753	KJ957584	-	-	-
**Pek4**	Indonesia	Pekanbaru	0.4220333	101.4367167	GPS	GQ377753	KJ957585	KJ957287	KJ957351	KJ957395
**Pek5**	Indonesia	Pekanbaru	0.4220333	101.4367167	GPS	GQ377753	KJ957586	-	-	-
**Pek7**	Indonesia	Pekanbaru	0.4220333	101.4367167	GPS	GQ377753	KJ957608	KJ957301	KJ957368	KJ957408
**Pek8**	Indonesia	Pekanbaru	0.4220333	101.4367167	GPS	GQ377753	KJ957609	KJ957302	KJ957369	KJ957409
**Bal2**	Indonesia	Bali	-8.509193	115.567178	GPS	GQ377753	KJ957561	-	-	-
**Bal4**	Indonesia	Bali	-8.509193	115.567178	GPS	GQ377753	KJ957562	-	KJ957359	KJ957401
**Bal5**	Indonesia	Bali	-8.509193	115.567178	GPS	GQ377753	KJ957563	KJ957295	KJ957360	KJ957402

**Bal6**	Indonesia	Bali	-8.509193	115.567178	GPS	GQ377753	KJ957564	-	KJ957361	KJ957403
**Sul1**	Indonesia	Sulawesi, Toletole stream	-2.5277333	121.1121	GPS	GQ377753	KJ957556	KJ957288	KJ957352	KJ957396
**Sul2**	Indonesia	Sulawesi, Toletole stream	-2.5277333	121.1121	GPS	GQ377753	KJ957557	KJ957316	KJ957382	KJ957421
**Sul3**	Indonesia	Sulawesi, Toletole stream	-2.5277333	121.1121	GPS	GQ377753	KJ957559	KJ957317	KJ957383	KJ957422
**Sul4**	Indonesia	Sulawesi, Toletole stream	-2.5277333	121.1121	GPS	GQ377753	KJ957560	-	-	-
**Sul5**	Indonesia	Sulawesi, Toletole stream	-2.5277333	121.1121	GPS	GQ377753	KJ957558	-	-	-
**Sul13**	Indonesia	Sulawesi	-2.816	121.58325	GPS	GQ377753	-	-	-	-
**SBP1**	Malaysia	Sg. Batu Pahat, Perlis	6.5085389	100.1766278	GPS	GQ377753	-	KJ957307	KJ957373	KJ957413
**SBP2**	Malaysia	Sg. Batu Pahat, Perlis	6.5085389	100.1766278	GPS	GQ377753	KJ957614	KJ957308	KJ957374	KJ957414
**SBP3**	Malaysia	Sg. Batu Pahat, Perlis	6.5085389	100.1766278	GPS	GQ377753	KJ957615	KJ957309	KJ957375	KJ957415
**SBP4**	Malaysia	Sg. Batu Pahat, Perlis	6.5085389	100.1766278	GPS	GQ377753	-	-	-	-
**SR01**	Indonesia	Sibreh, Aceh, Sumatra	5.4615972	95.3786139	GPS	GQ377752	-	-	-	-
**SR04**	Indonesia	Sibreh, Aceh, Sumatra	5.4615972	95.3786139	GPS	GQ377753	KJ957618	KJ957313	KJ957379	KJ957418
**SR08**	Indonesia	Sibreh, Aceh, Sumatra	5.4615972	95.3786139	GPS	GQ377753	KJ957591	KJ957314	KJ957380	KJ957419
**SR10**	Indonesia	Sibreh, Aceh, Sumatra	5.4615972	95.3786139	GPS	GQ377753	KJ957592	KJ957315	KJ957381	KJ957420
**LR5768**	Malaysia	Dungun	4.881381	103.375834	GPS	GQ377753	-	-	-	-
**LR5769**	Malaysia	Dungun	4.881381	103.375834	GPS	GQ377753	-	-	-	-
**LR5770**	Malaysia	Dungun	4.881381	103.375834	GPS	GQ377753	KJ957574	KJ957286	KJ957350	KJ957394
**LR5771**	Malaysia	Dungun	4.881381	103.375834	GPS	GQ377753	KJ957575	-	-	-
**LR5774**	Malaysia	Dungun	4.881381	103.375834	GPS	GQ377753	KJ957576	-	-	-
**LR5775**	Malaysia	Dungun	4.881381	103.375834	GPS	GQ377753	KJ957577	-	-	-
**LR5778**	Malaysia	Dungun	4.881381	103.375834	GPS	GQ377753	KJ957578	-	-	KJ957426
**LR5779**	Malaysia	Dungun	4.881381	103.375834	GPS	GQ377753	KJ957579	-	KJ957387	KJ957425
**LR5780**	Malaysia	Dungun	4.881381	103.375834	GPS	GQ377753	KJ957580	-	-	-
**LR5781**	Malaysia	Dungun	4.881381	103.375834	GPS	GQ377753	KJ957581	-	-	-
**LR5798**	Malaysia	Dungun	4.881381	103.375834	GPS	GQ377753	-	-	-	-
**LR6971**	Vietnam	Kien Giang province	10.30592	103.95361	GPS	GQ377753	KJ957594	KJ957320	KJ957388	KJ957428
**LR6972**	Vietnam	Kien Giang province	10.30592	103.95361	GPS	GQ377753	KJ957595	KJ957321	KJ957389	KJ957429
**LR6973**	Vietnam	Kien Giang province	10.30592	103.95361	GPS	GQ377753	-	KJ957322	KJ957390	KJ957430

**LR6974**	Vietnam	Kien Giang province	10.30592	103.95361	GPS	GQ377753	KJ957596	-	-	-
**LR6975**	Vietnam	Kien Giang province	10.30592	103.95361	GPS	GQ377753	KJ957597	-	-	-
**LR6976**	Vietnam	Kien Giang province	10.30592	103.95361	GPS	GQ377753	KJ957598	-	-	-
**LR6936**	India	Kalkotta	-	-	GE	GQ377753	-	KJ957319	-	KJ957427
**7001**	Cambodia	Near Andoung Tuek	11.20258	103.44572	GPS	GQ377753	-	KJ957289	KJ957353	-
**7002**	Cambodia	Near Andoung Tuek	11.20258	103.44572	GPS	GQ377753	KJ957600	KJ957290	KJ957354	KJ957397
**7003**	Cambodia	Near Andoung Tuek	11.20258	103.44572	GPS	GQ377753	KJ957599	KJ957291	KJ957355	-
**7005**	Cambodia	Near Andoung Tuek	11.20258	103.44572	GPS	GQ377753	KJ957601	-	-	-
**7008**	Cambodia	Near Andoung Tuek	11.20258	103.44572	GPS	GQ377753	KJ957602	-	-	-
**7020**	Vietnam	Kien Giang province	10.32995	103.96661	GPS	-	KJ957603	KJ957292	KJ957356	KJ957398
**7022**	Vietnam	Kien Giang province	10.32995	103.96661	GPS	-	KJ957604	KJ957293	KJ957357	KJ957399
**7023**	Vietnam	Kien Giang province	10.32995	103.96661	GPS	-	KJ957605	KJ957294	KJ957358	KJ957400
**7024**	Vietnam	Kien Giang province	10.32995	103.96661	GPS	-	KJ957606	-	-	-
