# Supplementary figures and images for: Plio-Pleistocene phylogeography of the Southeast Asian Blue Panchax killifish, Aplocheilus panchax
Source: PLoS One. 2017 Jul 25;12(7):e0179557. doi: 10.1371/journal.pone.0179557 (PMC5526567; doi:10.1371/journal.pone.0179557)

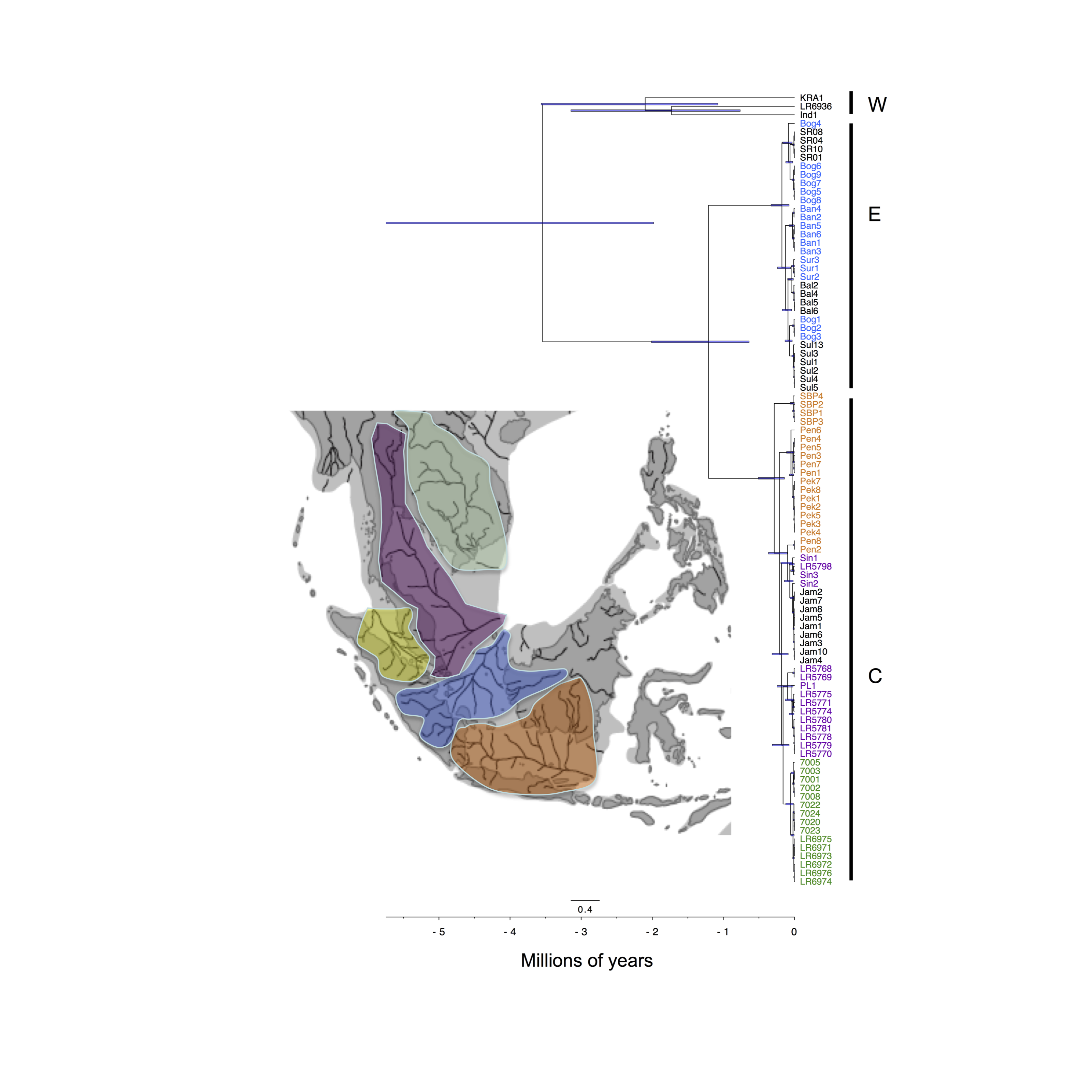

Supplement: S1 Fig — A time calibrated mitochondrial tree with 95% highest posterior densities. Southeast Asia’s palaeodrainage basins are coloured, as well as corresponding mitochondrial samples. Those samples that do not fall within a drainage basin, possibly as a result of stepping-stone dispersal, are left uncoloured. Western (W), Eastern (E) and Central (C) mitochondrial clades are also indicated. (TIF) [file pone.0179557.s002.tif]

a)

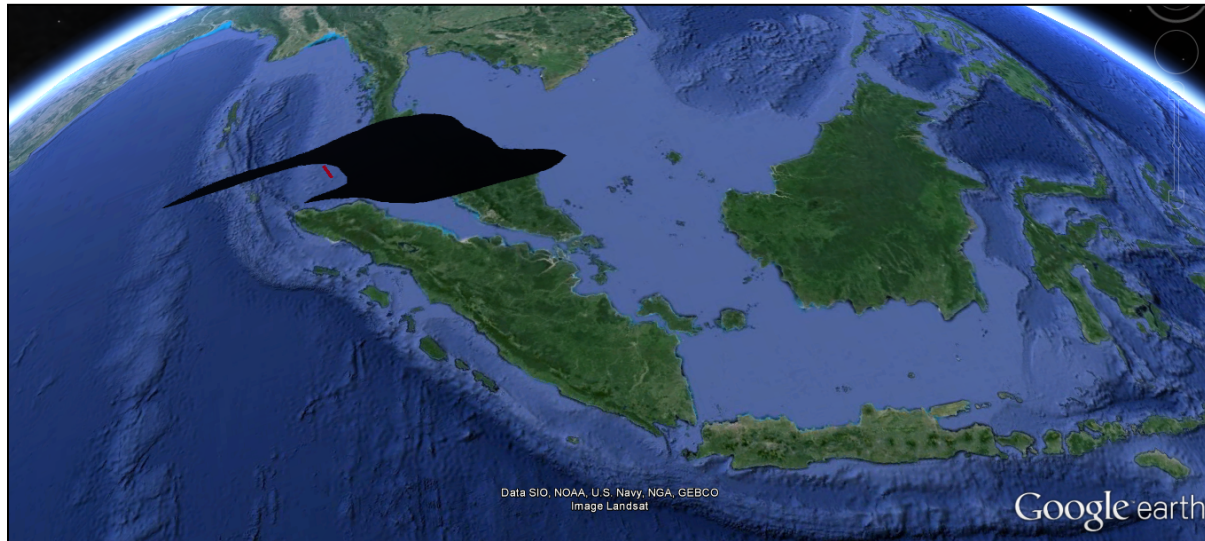

b)

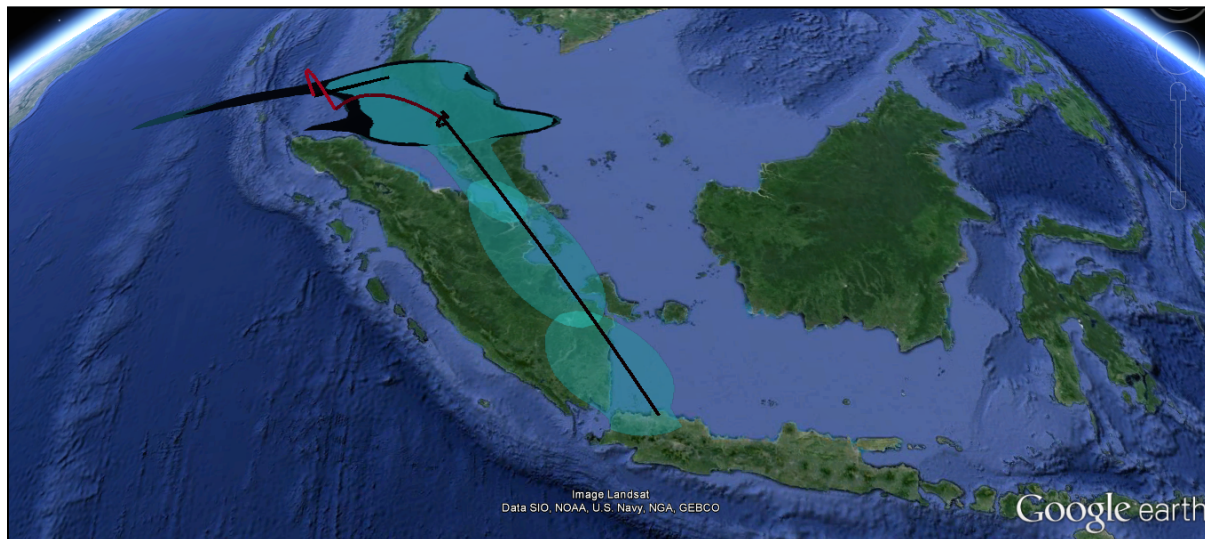

c)

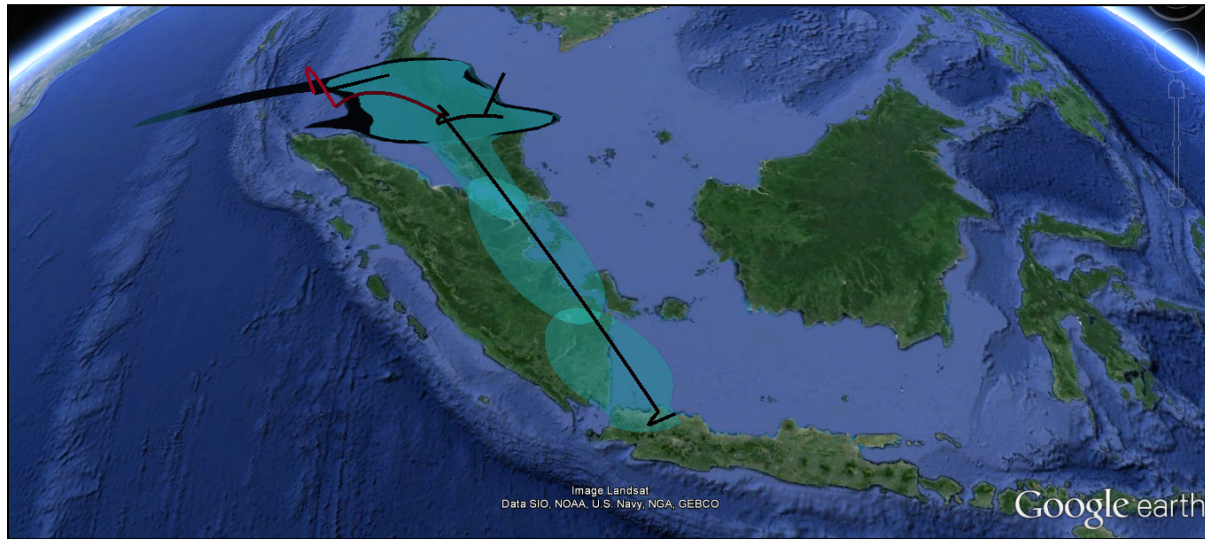

d)

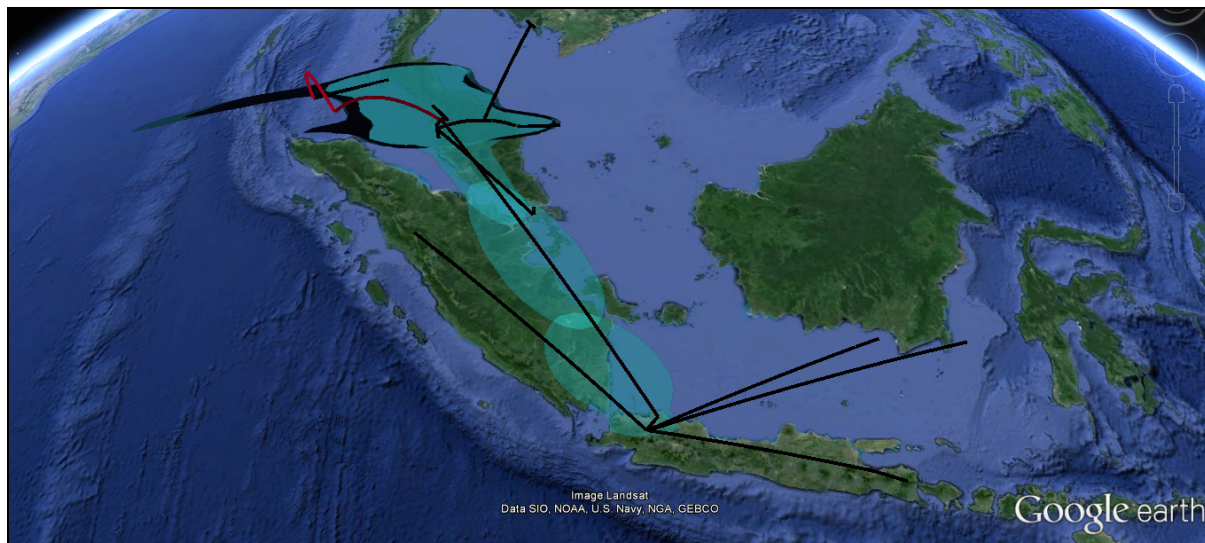

Supplement: S2 Fig — Map of SE Asia visualising dispersal timings of Aplocheilus panchax using molecular dating from the time calibrated mitochondrial tree (see Fig 2 in main text): a) 3.39Ma, b) 173,486ka, c) 114,987ka and d) 27,237ka. Black shaded areas indicate the confidence surrounding the ancestral locations, whilst blue shaded areas show 80% HPD. (PDF) [file pone.0179557.s003.pdf]
