# Supplementary material for: Plio-Pleistocene phylogeography of the Southeast Asian Blue Panchax killifish, Aplocheilus panchax
Source: PLoS One. 2017 Jul 25;12(7):e0179557. doi: 10.1371/journal.pone.0179557 (PMC5526567; doi:10.1371/journal.pone.0179557)
Supplement: S3 Fig — Median-joining nuclear allele networks for Aplocheilus panchax for three anonymous nuclear markers: a) AP44, b) AP50, and c) AP70. Colours correspond to geographic location, node size is proportional to allele frequency and numbers indicate the number of mutations. (PDF) [file pone.0179557.s004.pdf]

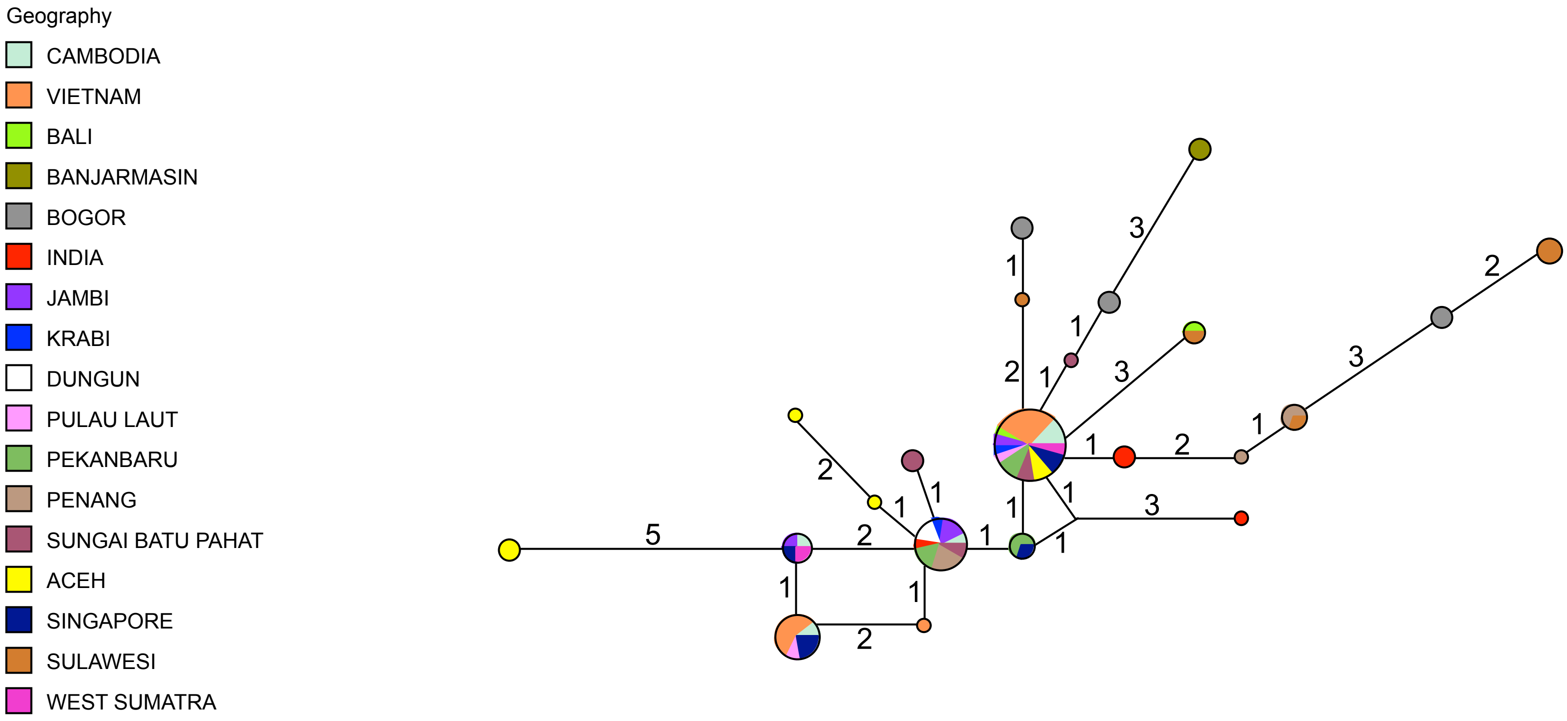
Geography
CAMBODIA
VIETNAM
BALI
BANJARMASIN
BOGOR
INDIA
JAMBI
KRABI
DUNGUN
PULAU LAUT
PEKANBARU
PENANG
SUNGAI BATU PAHAT
ACEH
SINGAPORE
SULAWESI
WEST SUMATRA
5
2
1
1
2
1
1
2
1
1
1
2
1
1
3
3
1
2
1
3
3
2
1
3
1
1
1

a)

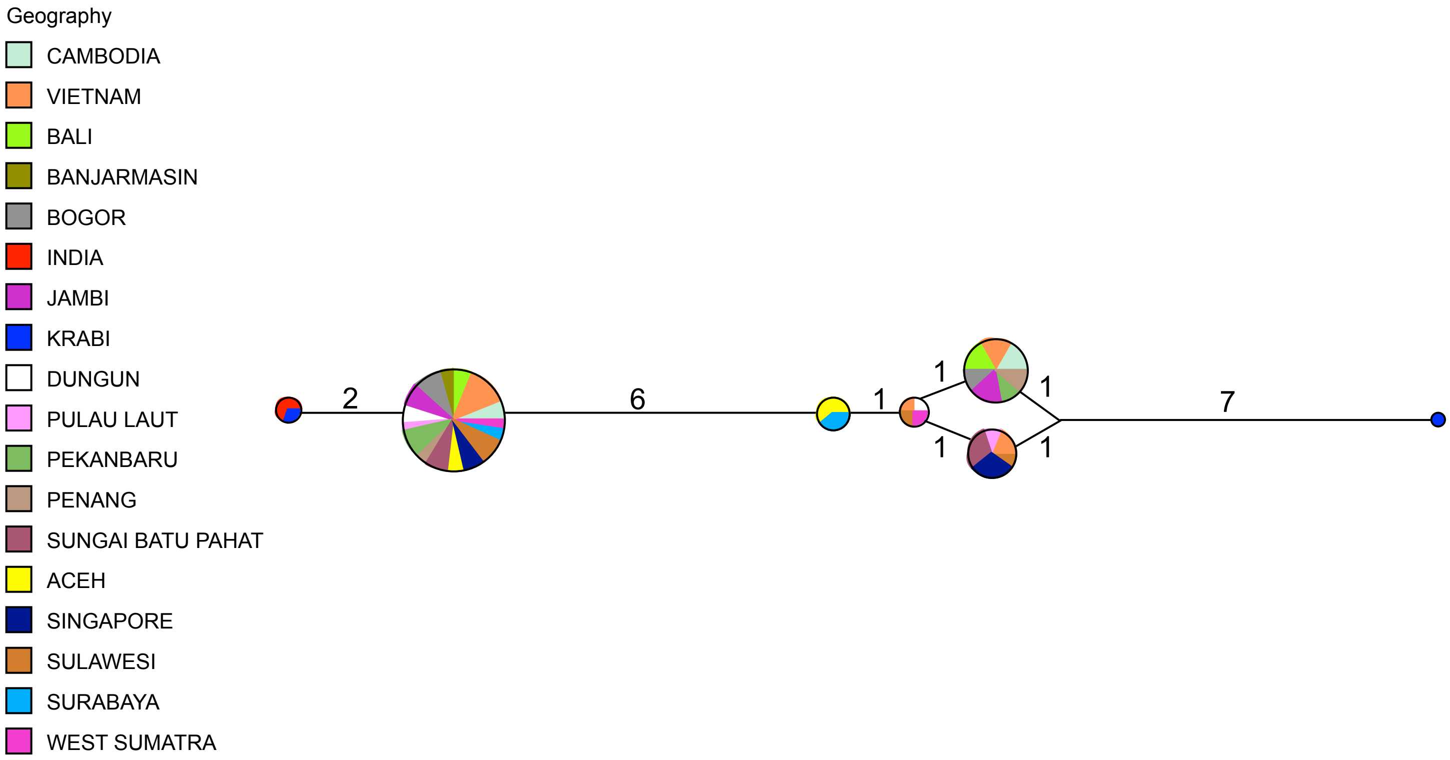
Geography
CAMBODIA
VIETNAM
BALI
BANJARMASIN
BOGOR
INDIA
JAMBI
KRABI
DUNGUN
PULAU LAUT
PEKANBARU
PENANG
SUNGAI BATU PAHAT
ACEH
SINGAPORE
SULAWESI
SURABAYA
WEST SUMATRA
2
6
1
1
1
1
1
7

b)

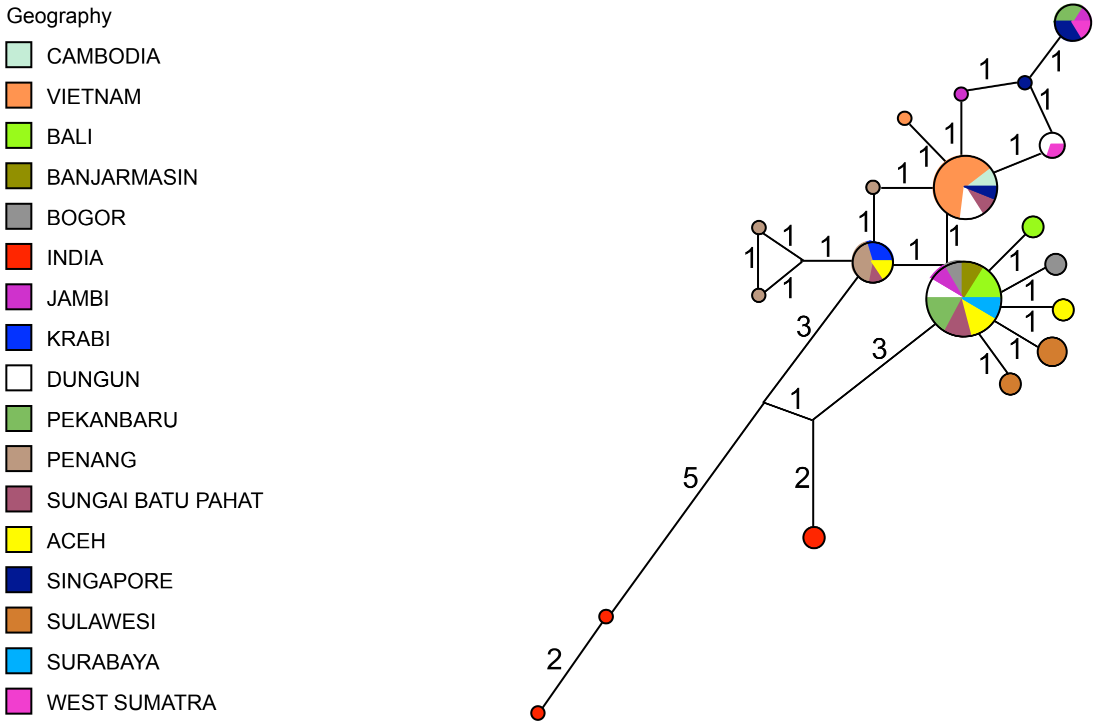
Geography
CAMBODIA
VIETNAM
BALI
BANJARMASIN
BOGOR
INDIA
JAMBI
KRABI
DUNGUN
PEKANBARU
PENANG
SUNGAI BATU PAHAT
ACEH
SINGAPORE
SULAWESI
SURABAYA
WEST SUMATRA

c)
